# Parallel gold enhancement of quantum dots 565/655 for double-labelling correlative light and electron microscopy on human autopsied samples

**DOI:** 10.1038/s41598-022-09849-8

**Published:** 2022-04-12

**Authors:** Miho Uematsu, Kyohei Mikami, Ayako Nakamura, Ryosuke Takahashi, Takanori Yokota, Katsuiku Hirokawa, Toshiki Uchihara

**Affiliations:** 1 Structural Neuropathology, Tokyo Metropolitan Institute of Medical Science, Tokyo, Japan; 2 Histology Center, Tokyo Metropolitan Institute of Medical Science, Tokyo, Japan; 3 Department of Neurology, Faculty of Medicine, University of Kyoto, Kyoto, Japan; 4 Department of Immunology and Genomics, Graduate School of Medicine, Osaka Metropolitan University, Osaka, Japan; 5 Neurology Clinic With Neuromorphomics Laboratory, Nitobe-Memorial Nakano General Hospital, Tokyo, Japan; 6 Department of Pathology, Nitobe-Memorial Nakano General Hospital, Tokyo Medical and Dental University, Tokyo, Japan; 7 Department of Neurology and Neurological Science, Tokyo Medical and Dental University, 1-5-45 Yushima, Bunkyo-ku, Tokyo, Japan

**Keywords:** Biological techniques, Biotechnology, Neuroscience, Structural biology, Anatomy, Nanoscience and technology, Optics and photonics

## Abstract

Cadmium selenide quantum dots (QDs) are fluorescent and electron-dense nanoparticles. When used as reporter of immunolabeling, this dual visibility is essential for direct comparison of its fluorescent signals on light microscopy (LM) and their ultrastructrual counterparts on electron microscopy (EM) as correlative light and electron microscopy (CLEM). To facilitate EM recognition, QDs on EM grid were gold enhanced, which increased their size and electron density. On histological sections as well, gold-enhanced QDs, used as a reporter of immunolabeling, were easily recognized on EM. Because target structures are visible on bright field microscopy, gold enhancement facilitated trimming the target structures into final EM sections. Furthermore, gold enhancement of rod-shaped QD655 on EM grid was accentuated on their tips while spherical QD565 was gold-enhanced as sphere in contrast. This EM distinction was evident on histological sections where QD565 (green fluorescence) and QD655 (red fluorescence) were used as a reporter pair for double immunolabeling. Double-labeled immuno-fluorescent images, initially captured before EM processing, are now compared with their respective immuno EM counterparts. Specific labeling of each epitope was corroborated by mutual comparison between LM and EM. Although fluoronanogold may be a candidate reporter partner with QDs for gold-enhanced, double-labeling CLEM, its limited penetration into fixed tissue hampers universal use for thick histological sections. Gold-enhancement of QD immunolabeling, now expanded to double-labeling CLEM for human brain samples, will pave the way to translate molecular events into ultrastructural morphopathogenesis in situ.

## Introduction

Immunolocalization of molecules in association with their target structures is important for understanding disease processes and tissue diagnostics. These immunolabeling signals are detected through reporters customized for different microscopes, such as fluorochromes for light microscopy (LM) and electron-dense particles for electron microscopy (EM). Because tissue preparation for LM and that for EM are quite different, it is necessary for LM/EM comparison to divide the specimen into halves, dehydrate one half and seal it in epoxy resin for EM, and use the other half for LM without drying it to avoid fading of fluorescence. Even if the divided specimen pair showed similar lesions, the comparison between LM and EM would be inevitably indirect. Use of dual modality reporter with high contrast in both LM and EM specimens may provide a way to observe the same target in LM and EM. If specimens suitable for both fluorescence and EM are prepared and the two imaging modalities are directly compared, the disadvantages of each can be compensated for and the gap between LM and EM images can be closed. This immediate comparison between LM and EM is called correlative light and electron microscopy (CLEM), which was first proposed in the 1990s and has since developed through advanced technologies in various fields^1,2^. Various LM observation instruments^3,4^, EM observation instruments^5^, and integrated instruments^6,7^ have been used in CLEM . There are also many options for sample preparation methods^8,9^, antibodies^10^, labels (dual-modality probes such as quantum dots (QDs)^11–13^, fluorescent dye-bound nanogolds^14^, fluorescent gold or platinum nanoclasters^15,16^, nanodiamonds^3^, and fixation resistant fluorescence^17^, etc.) and fiducial marks^18^. The best method to choose depends greatly on the type of sample under investigation. The quality of the fluorescent images and ultrastructures obtained also depends on the type of sample that can be used. Cadmium selenide (CdSe) QDs are electron-dense, fluorescent nanocrystals. When used as a reporter for immunolabeling, QDs emit detectable fluorescence in LM and are recognized as electron-dense particles in EM^11–13^. However, QD labels are colorless in bright-field LM, and QD fluorescence is quenched when fixed with osmium tetroxide (OsO_4_). Therefore, it is very difficult to reposition the fluorescent targets identified in LM during EM preparation. We have circumvented these difficulties by placing additional landmarks by microlaser around LM-identified target during LM observation prior to EM preparation^19–21^ for desired trimming. Even after LM-identified targets were successfully retrieved on the final EM sections, electron density of QDs may not be sufficiently intense. It is possible to confirm the identity of labeling QDs by analyzing the elemental composition (cadmium and selenium) by energy dispersive X-ray (EDX) analysis^20^. Although this complex protocol is feasible and reliable, it requires microlaser to landmark the QD-positive target and special equipment for EDX analysis.

Here, using human autopsy brain sections, we show that QD-labeled target structures in the tissues can be visualized with LM by gold enhancement, and that different types of QDs have different gold enhancement modes and can be identified by immunoEM.

## Methods

### Ethics approvals

This study was approved by the research ethics committee of Tokyo Metropolitan Institute of Medical Science (Permission number: 16-25). The research use of autopsied brains was conducted in compliance with Japan’s Postmortem Examination and Corpse Preservation Act and with informed consent from the next of kin of all subjects. All methods were carried out in accordance with relevant guidelines and regulations (Declaration of Helsinki).

### Gold enhancement of the QDs on nickel grids

**Figure 1:**
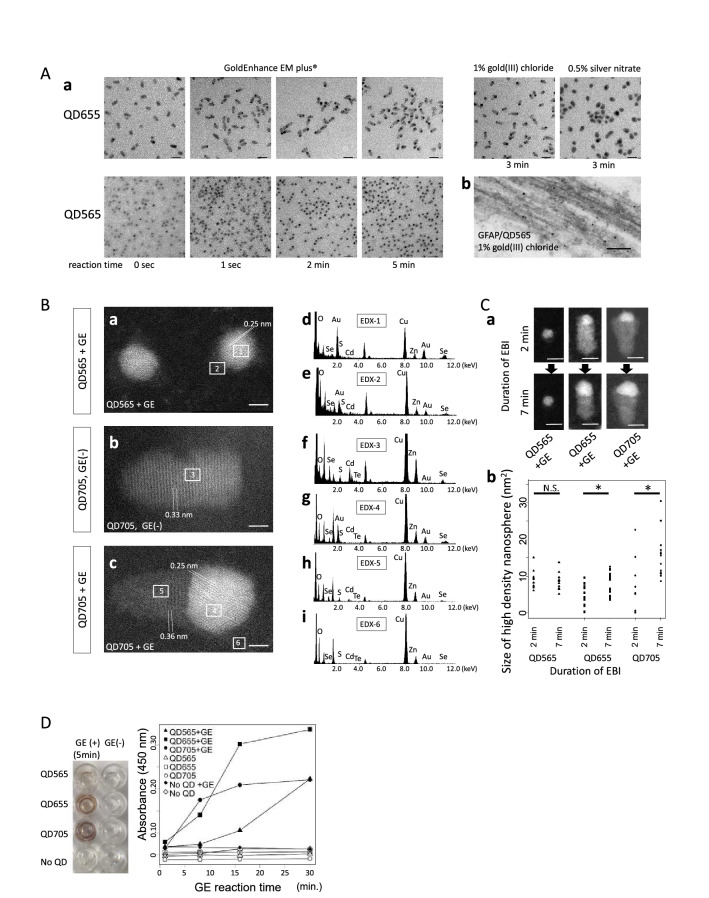
Gold enhancement of QD particles intensifies their electron density by progressive accumulation of gold. **(A)** (a) QD655 and QD565 particles before and after gold enhancement for 1 s, 2 min, and 5 min using GoldEnhance EM plus® are shown. Alternatively, Gold enhancement using 1% gold (III) chloride solution for 3 min, or silver enhancement using 0.5% silver nitrate solution for 3 min was also performed on QD655 particles. Enhanced EM contrast of the whole particles of QD565 and the tips of rod-shaped QD655 was obtained after gold enhancement. Bars: 20 nm. (b) A brain section labeled for GFAP/QD565 was gold enhanced using 1% gold (III) chloride solution for 3 min. Bar: 100 nm. **(B)** High-angle annular dark field scanning transmission electron microscope (HAADF-STEM) image and EDX spot analysis of gold enhanced QD565 (**a**,**d**,**e**), native QD705 (**b**,**f**), and gold enhanced QD705 particles (**c**,**g**,**h**,**i**) on a nickel grid. Numbered rectangles in the EM images correspond to the areas for EDX spot analysis. Gold enhanced QD705 consisted of two different crystalloid structures with different interplanar spacing. GE: gold enhancement. Bars: 2 nm. **(C)** (a) Gradual increase in size of high contrast area during continuous STEM observation. EBI: electron beam irradiation. (b) Sizes of high density nanospheres on QD565, 655, and QD705 after GE was obtained 2 min and 7 min after continuous EBI. * P < 0.05 (Welch’s two sample t-test). Bars: 5 nm. **(D)** Change in color and absorbance (450 nm) of quantum dot (QD) solution by gold enhancement in the well. *GE* gold enhancement.

To monitor the influence of gold enhancer on QD particles, 1.5 μl of QD565, QD655, or QD705 solutions (1:30 dilution in distilled water, Invitrogen, Thermo Fisher Scientific, MA, USA) were placed onto the formvar coated nickel grids, and the grids were air-dried. GoldEnhance EM plus (Nanoprobes, NY, USA) is an autometallographic enhancer which catalytically deposits gold ions in solution onto the nanoparticle as metallic gold. 40 μl droplets of the reagent mixture of GoldEnhance EM plus were prepared according to the manufacturer’s protocol, and placed on the Parafilm (Bemis Company, Inc, WI, USA) strip. The grids were immersed in the GoldEnhance EM plus droplets for various time (1 s, 2 min, or 5 min) (Fig. 1Aa). Alternatively, the grids were immersed in 1% gold (III) chloride solution or 0.5% silver nitrate solution diluted in distilled water for 3 min (Fig. 1Aa). The grids were subsequently rinsed with distilled water, air-dried, and observed under EM.

### Gold enhancement of the QD in microtiter plates

To quantify bright-field coloration after gold enhancement of QDs, gold enhancement reaction was performed in the microtiter plates and absorbance was measured. 10 μL goat anti-mouse IgG antibody (1:500 / phosphate-buffered saline (PBS)) was immobilized in the wells of microtiter plates (Nunc-Immuno MicroWell 96-well Plates, Thermo Fisher Scientific, MA, USA) overnight at 4 °C. Subsequently, the wells were incubated with 100 μL normal mouse serum (1:500/PBS) for 1 h at room temperature (RT). Some of the wells were further incubated with biotinylated goat anti-mouse IgG (1:1000/PBS) for 1 h, and ABC complex (1:50/PBS, Vector Laboratories, CA, USA) for 1 h at RT, for the subsequent reaction with streptavidin. Wells were washed three times with PBS containing 0.05% Triton X-100 between each step. Subsequently, after washing three times with 0.1 M Tris HCl, QD565-conjugated goat anti-mouse IgG (1: 200/0.1 M Tris HCl, Invitrogen, CA, USA), QD655-conjugated streptavidin (1: 200/0.1 M Tris HCl, Invitrogen, CA, USA), QD705-conjugated goat anti-mouse IgG (1: 200/0.1 M Tris HCl, Invitrogen, CA, USA) were added to each well and allowed to stand for 1 h. After washing three times with 0.1 M Tris HCl, 100 μL GoldEnhance EM plus was added, and reaction was carried out for up to 30 min. Absorbance was measured at 450 nm using Biotrak II (GE healthcare, IL, USA) at 1, 8, 16, and 30 min.

### Tissue preparation from autopsied human brain

The autopsied brains were routinely immersion-fixed in 4% formalin for 4 weeks. Tissue blocks of the temporal lobes were washed in 0.1 mol/L phosphate buffer (PB) and were cryoprotected with 15% sucrose/PB overnight and then in 30% sucrose/PB overnight at 4 °C. The tissue was then immersed in Optimal Cutting Temperature compound (Sakura Finetek Japan, Tokyo, Japan) and rapidly frozen on the stage of a carbon dioxide freezer kept below −10 °C. 26 μm-thick floating sections were prepared by cutting the tissue on a sliding microtome equipped with a freezing stage below −10 °C for double labeling toward phospho-tau (Ser202, Thr205) and glial fibrillary acidic protein (GFAP). Protocols for floating sections are summarized in Table 1.

**Table 1:** A summary of QD-CLEM protocols for floating sections.

Procedure	**CLEM (floating sections)**
Tissue preparation	Immersion fixation of human autopsied brain Coronal sectioning at 1 cm intervals Cryoprotection Rapid freezing on the stage of a carbon dioxide freezer Floating sections
LM preparation	Blocking Primary antibodies incubation **Secondary antibodies incubation (QD/QD or QD/nanogold)**
LM imaging	Fluorescence and bright-field imaging Primary post-fixation by 1% glutaraldehyde **Gold enhancement**
EM preparation	Secondary Post-fixation by 1% OsO_4_ Dehydration Epon embedding between Aclar films Bright-field imaging Attachment of epon cylinder to the ROI and polymerization Target-oriented trimming Ultrathin sectioning Post-staining of EM grids with uranyl acetate and lead
EM imaging	EM observation and EDX analysis

### Dual immunolabelling with QDs or QDs/fluoronanogold

**Figure 2:**
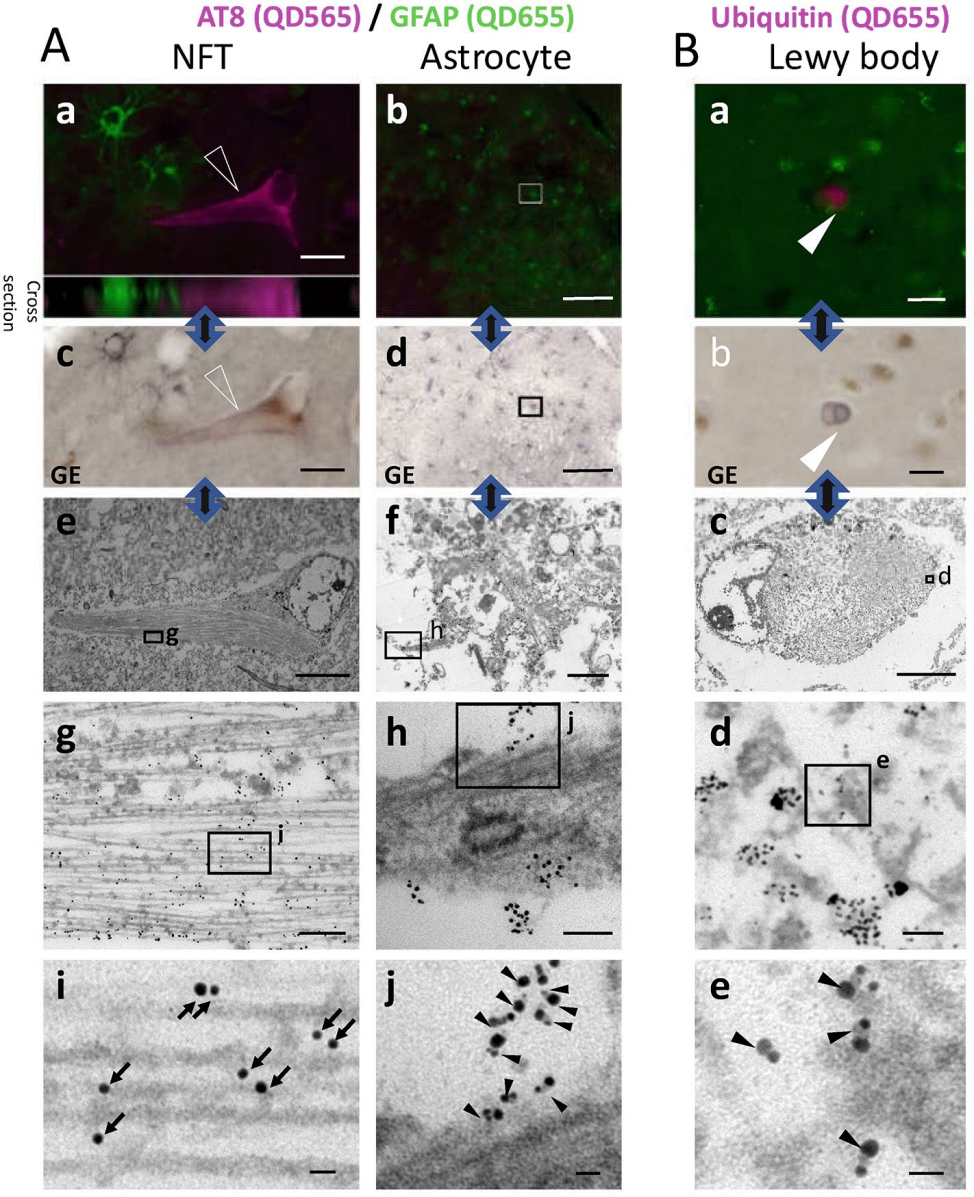
EM distinction of QD565 for AT8 and QD655 for GFAP after gold enhancement and their LM counterparts on double immunolabeled section. **(A)** A section from the autopsied brain was double immunolabeled for AT8/QD565 (magenta) and GFAP/QD655 (green). (a) Extended focus image (EFI) of an NFT (open triangle) and astrocytes and its cross-sectional reconstruction (20 μm thick). Fluorescent signals from QD565 and QD655 were similarly intense throughout the entire depth of the section. (b) EFI of astrocytes on the same section at different site. An astrocyte enclosed in the rectangle was targeted for subsequent EM observation. (c,d) Bright-field LM counterpart of targets in (a,b) after gold enhancement and post-fixation. QD565 and QD655 labeled structures are darkened. (e–j) EM images corresponding to the LM images, after gold enhancement of the section. The areas enclosed by rectangles correspond to the images just below. While the filaments in NFT (e,g,i) was labeled with high contrast spherical QD565 (arrow), the glial fibers of the astrocyte (f,h,j) was labeled with rod-like QD655, which showed high contrast nanospheres deposited at the tips of the low contrast QD655 nanorods (filled triangle). The QD565 (in i, arrow) and the QD655 label (in j, filled triangle) are readily distinguishable from each other by their characteristic shapes after gold enhancement. Bars in a: 20 μm, b: 100 μm, c: 20 μm, d: 100 μm, e: 10 μm, f: 2 μm, g: 200 nm, h: 100 nm, i and j: 20 nm. **(B)** Another section of the same autopsied brain was labeled with ubiquitin/QD655 (magenta). A fluorescence image of a Lewy body (a, open triangle) were directly compared with bright-field LM image (b) and EM image (c–e) after gold enhancement. Loose and short ubiquitin-positive fibers of a Lewy body was labeled by rod-like QD655 (e, filled triangle), which showed high contrast nanosphere deposits at the tips of the nanorods. Bars in a and b: 20 μm, c: 5 μm, d: 100 nm, e: 20 nm.

26 μm-thick floating sections were washed with PBS, blocked for 30 min in 5% normal goat serum/0.05% NaN_3_/PBS, and incubated with the mixture of anti-phospho-tau (Ser202, Thr205) antibody AT8 (1:1000, mouse monoclonal, Thermo Fisher Scientific, MA, USA), and anti-GFAP (1:100, rabbit polyclonal, DAKO, Agilent Technologies, CA, USA), diluted in the blocking buffer, for approximately 96 h at 4 °C. For single immunolabeling for ubiquitin, anti-ubiquitin (1:100, rabbit polyclonal, DAKO, Agilent Technologies, CA, USA) was used as primary antibody. The sections were washed with PBS, incubated with biotinylated secondary anti-rabbit antibody (1:200, BA-1000, Vector Laboratories, CA, USA) and AT8 (1:1000), diluted in PBS, for 3 days. After washing three times with PBS, sections were subsequently incubated with avidin biotinylated enzyme complex (1:200, Elite ABC, Vector Laboratories, CA, USA) diluted in PBS for 1 h, to enhance the interaction between biotinylated secondary antibody and streptavidin. Sections were washed with Tris-buffered saline (TBS). AT8 antibody was selectively labeled with QD565 conjugated with F(ab')2 anti-mouse IgG (H + L) (1:50, Invitrogen, CA, USA), and anti-GFAP antibody or anti-ubiquitin antibody was selectively labeled with either QD655 conjugated with streptavidin (1:50, Invitrogen, CA, USA) (Fig. 2A) or Alexa488-1.4 nm nanogold conjugated with streptavidin (1:50, Nanoprobes, NY, USA) (Supplementary Figs. 3, 4), diluted in TBS for 90 min in the dark. After washing with TBS, the sections were put in a glass container filled with washing buffer, and scooped up using the face of the glass slide. The glass slide was tilted to remove droplets of buffer and glycerol was added gently. A cover glass was placed and the edge of the cover glass was sealed. We avoided the use of PAP pen, prolonged incubation in PBS at low concentration, or anti-fade reagent containing *p*-phenylenediamine, to avoid quenching of QD fluorescence.

### Light microscopic detection with a virtual slide system

The immunolabeled free-floating sections were mounted on glass slides and observed using a virtual slide system VS120 (Olympus, Tokyo, Japan) equipped with UPLSAPO objective lens (10, 20, and 40 times dry objectives, and a 60 times water-immersion objective, Olympus, Tokyo, Japan), a 130 W U-HGLGPS light guide-coupled illumination system (Olympus, Tokyo, Japan), a triple-band dichroic mirror unit U-DM3-DA/FI/TX (Olympus, Tokyo, Japan, 480–500 nm band-pass excitation filter, 505 nm dichroic mirror, 515–540 nm band-pass emission filter) (for Alexa 488), a filter set AT-Qdot565 (Chroma Japan Corp., Yokohama, Japan, 400–450 nm band-pass excitation filter, 485 nm dichroic mirror, 550–580 nm band-pass emission filter) installed in the filter cube U-MF2 (Chroma Japan Corp., Yokohama, Japan) (for QD565), a mirror unit U-DM3-QD655 (Olympus, Tokyo, Japan, 410–460 nm band-pass excitation filter, 505 nm dichroic mirror, 640–670 nm band-pass emission filter) (for QD655), a sensitive cooled charge-coupled device camera (ORCA-R2 C10600-10B, Hamamatsu Photonics, Shizuoka, Japan), and VS-ASW software (Olympus, Tokyo, Japan). Serial snapshots of immunofluorolabeled sections on motorized stage were captured by VS120 through 10 times objective lens in separate fluorescence channels, and put together to make a seamless broad image, covering the whole section. Areas of particular interest were also captured with 60 times water-immersion objective lens with multiple z-stacks at 1 μm intervals, or with 20 times objective lens at 2 μm intervals. The image size of each snapshot was 1376 pixels (horizontal) × 1038 pixels (vertical), at 0.645 μm/pixel with 10 times objective lens, 0.323 μm/pixel with 20 times objective lens, and 0.107 μm/pixel with 60 times objective lens. Cross sectional views were obtained by the ImageJ volume viewer plugin (Barthel, K. U., version 2.01, https://imagej.nih.gov/ij/plugins/volume-viewer.html).

### Gold enhancement of QD labels on tissue sections and electron microscopy (EM) preparation

After the fluorescent virtual slide images were taken, the floating sections were gold enhanced and prepared for EM as follows. First, the cover glass of the floating sections was detached in TBS, and the sections were washed with TBS and post-fixed with 1% glutaraldehyde in TBS for 15 minutes at room temperature. After washing in TBS with 50 mM glycine, 1% bovine serum albumin in TBS, and distilled water, floating sections were immersed for 5 min in a 40 μl droplet of GoldEnhance EM plus reagent mixture on Parafilm, prepared according to manufacturer’s protocol. Sufficient bright-field coloration was obtained. On the other hand, gold enhancement was also attempted on another section by immersing the section in a 40 μl droplet of 1% gold (III) chloride solution for 5 min (Fig. 1Ab). However, 1% gold (III) chloride solution did not sufficiently alter the color of the labeling in bright field. Therefore, GoldEnhance EM plus was selected for further observation in tissue sections. After rinsing with distilled water, the sections were mounted with glycerol and examined with a virtual slide system VS120 equipped with a VC50 camera (Olympus, Tokyo, Japan) through a UPLSAPO 10× dry objective. The image size of each snapshot was 1376 pixels (width) × 1038 pixels (height), at 0.691 μm/pixel. The sections were then postfixed in 1% osmium tetroxide in 0.1M PB for 1.5 h at 4 °C, followed by dehydration in graded ethanol series (50%, 70%, 80%, and 90%) each for 5 min, and 100% ethanol for 30 min, respectively, three times. The sections were immersed in 100% propylene oxide and gently shaken for 15 minutes at room temperature. The 100% propylene oxide was then replaced with new 100% propylene oxide, and the same operation was performed a total of three times for 15 minutes each. The sections were infiltrated first with a 1:1 mixture of propylene oxide and epon (Epon 812, Taab, Berks, England) for 1 h, and then with pure epon for 1 h at room temperature. The floating sections were then placed between aclar films (Honeywell, NC, USA) with a sufficient amount of pure epon and heated in an oven at 65 °C for 3 days, allowing the resin to polymerize while the sections are stretched, as described previously^20^. Using VS120, LM images of the sections infiltrated with epon between the aclar films were taken to obtain a bright-field tiled image around the target. The target was then located under a stereomicroscope and this area was cut out along with the aclar film using scissors or a safety razor blade. The aclar film can be easily peeled off. On a glass slide, a gelatin capsule filled with epon was invertedly placed on top of the resin section and polymerized at 65°C for at least 48 hours. The polymerized capsule block was detached from the glass slide while heated on a hot plate^20^.

Upon removal, the tissue was attached to the resin cylinder. The final trimming of the resin cylinder around the target was guided by enhanced QD labeling visible under a stereomicroscope. Ultrathin sections of 70 nm were made and placed on formvar coated grids. Ultrathin sections were post-stained with uranyl acetate for 25 min and lead for 7 min and observed by EM.

### EM observation and energy-dispersive X-ray (EDX) analysis

In order to confirm the presence of gold on the QD labelling on the sections after the gold enhancement, an energy spectrum of the metals was obtained on the EM sections. Ultrathin sections on EM grids were observed under a TEM(JEM-1400, JEOL, Tokyo, Japan) or a STEM , (Hitachi HD-2700, Hitachi High Technologies Corporation, Tokyo, Japan). Bright-field and high-angle annular dark-field (HAADF) STEM images were obtained using HD-2700, a 200 kV spherical aberration (Cs)-corrected STEM with secondary electron (SE) detector (Hitachi High Technologies Corporation, Tokyo, Japan). The STEM was also equipped with solid angle 100 mm^2^ silicon drift detector (SDD) Octane T Ultra (EDAX, AMETEK Inc., PA, USA), which is an elemental analysis instrument that uses characteristic X-rays from a specimen and distinguish different elements based on their energy spectra. Bright-field and HADDF-STEM images with 1280 × 960 pixels were acquired with an incident beam of 0.3 nm and a current of 0.5 nA for a frame time of 18 s per image (15 μs/pixel). EDX spot analyses were performed with an incident beam size of 0.3 nm and a current of 0.5 nA. The acquisition time of the EDX single point analysis was up to a few tens of seconds and varied depending on the specimen. In the EDX mapping, the EDX analysis was performed with the image resolution of 128 × 100 pixels, at 30,000 to 1,600,000 times magnification, and the total acquisition time was up to 12 min. Pixels containing peaks of different elements were displayed separately in different pseudo-color channels.

### Image analysis

EM observations of gold-enhanced QDs showed that high contrast nanospheres were gradually generated and became larger on the QD particles as electron beam irradiation (EBI) for EM was continued, as described in the later sections. Therefore, the size change of high contrast nanospheres during EM was evaluated by image analysis. ADF-STEM images of a fixed range were obtained 2 min and 7 min after the start of continuous EBI. ImageJ (National Institutes of Health, Maryland, USA) was used to binarize these ADF-STEM images to highlight the high-contrast nanospheres. The images of QD705 and QD655 were binarized with a grayscale value of 175 as the threshold, and the images of QD565 were binarized by IsoData algorithm. The sizes of the high contrast areas as well as their X–Y coordinate in the images were enumerated automatically by particle analysis program of ImageJ. On images 7 min after EBI, we defined particles with more than 3.40 nm^2^ area as nanospheres. Corresponding QDs were identified in the image after 2 min from the start of EBI by their XY coordinates, and the area of the high contrast portions were obtained. The differences of the nanosphere sizes between 2 and 7 min after EBI were statistically analyzed as below. Next, to highlight both the high contrast nanospheres and surrounding minute particles on the ADF-STEM images, the images of QDs were binarized with the gray scale value 150, and high contrast portions were highlighted with pseudocolored yellow (Supplementary Fig. 1B).

### Statistical analysis

All statistical analyses were performed using software R (version 3.6.0, R Foundation for Statistical Computing, Vienna, Austria.). To find the differences in the means of sizes of high-contrast nanospheres between samples, Welch’s two sample t-test was performed (Fig. 1C). P < 0.05 was considered significant.

## Results

### Gold enhancement of QD particles increases their EM visibility and sphere-shaped and rod-shaped QD particles are still distinguishable based on the selective gold nucleation at the tip of rod-shaped QDs

In immuno EM using QD labeling, the electron density of QD particles on EM sections is not always adequately highlighted against underlying ultrastructures stained by heavy metal. To see if the standard gold enhancement protocol improves the EM visibility of QD particles, spherical QD565 (CdSe core–ZnS shell, diameter ~ 5 nm), rod-shaped QD655 (CdSe core–ZnS shell, diameter ~ 15 nm) and rod-shaped QD705 (CdSeTe core–ZnS shell, ~ 20 nm diameter) particles were gold enhanced on EM grids and observed by EM (Fig. 1Aa**,** Supplementary Fig. 1A). Spherical QD565 was homogeneously replaced with the electron dense material, while deposition of highly electron dense material was seen only at the tip of rod-shaped QD655 and QD705 (Fig. 1Aa, Supplementary Fig. 1A). HAADF-STEM revealed gold-enhanced QD565 as a high-contrast particle (Fig. 1Ba), with interplanar spacing of 0.25 nm; gold peaks were detected on EDX (Fig. 1Bd,e). On the other hand, native QD705 particle without gold enhancement demonstrated an overall low contrast with interplanar spacing of 0.33 nm (Fig. 1Bb),and Cd and Se peaks were detected by EDX (Fig. 1Bf). The gold-enhanced QD705 exhibited a hybrid of two different crystalloid structures with different interplanar spacing (Fig. 1Bc). EDX spot analysis of the gold-enhanced QD705 showed gold peaks in the high electron density material (Fig. 1Bg), and Cd and Se peaks in the remaining low-contrast parts of the particle (Fig. 1Bh). Background did not show gold peaks by EDX (Fig. 1Bi). The diameters of the gold deposit at the tips of rod-shaped QD655 and QD705 gradually increased with prolongation of the gold-enhancement reaction time (Fig. 1Aa) and subsequent electron irradiation time (Fig. 1C, Supplementary Fig. 1B, Supplementary video), while the size of the gold precipitates in QD565 did not change significantly when the gold-enhancement reaction time or electron irradiation time was extended (Fig. 1Aa,C, Supplementary video).

Next, in order to clarify whether QDs are colored in the bright field due to gold enhancement, we performed gold enhancement of QDs in microtiter plates and monitored chronological increase in coloration by measuring absorbance. The results showed that the gold-enhanced QDs were colored in the bright field and had increased absorbance, and such coloration occurred only in the presence of both QDs and the gold enhancer (Fig. 1D). Later, this coloration was used to detect gold enhanced QDs in tissue sections.

These results indicate that the standard gold enhancement method increases the electron density of QD particles. Furthermore, the distinction between spherical and rod-shaped QDs was possible even after gold enhancement, as there was uniform nucleation around the spherical QDs, whereas there was selective nucleation at the tips of the rod-shaped QDs. In addition, the fact that the gold-enhanced QDs were colored in the bright field helped in recognizing the QD labels in the LM.

### Double labeling CLEM with QD565/QD655: clear distinction after gold enhancement

To see if gold-enhanced QD labeling can be applied to CLEM of the tissue sections, we immunolabeled a human autopsied brain sample with Alzheimer pathology. In Alzheimer disease, abnormal phospho-tau aggregates as neurofibrillary tangles (NFTs) and neuropil threads (NTs) in the brain, which is one of the pathological features of the disease^22–27^. We immunolabeled 26 μm-thick free-floating sections of formalin-fixed temporal lobe from an autopsied brain with Alzheimer pathology and concomitant Lewy body pathology (an 80-year-old male, Braak’s NFT stage III/neuritic plaque score A, Supplementary Table 1)^23,28^, using anti-phospho-tau antibody (AT8)/QD565 (CdSe–ZnS nanosphere, diameter ~ 5 nm, emission peak 565 nm, pseudocolored magenta), and anti-glial fibrillary acidic protein (GFAP) antibody/QD655 (CdSe–ZnS nanorod, diameter ~ 15 nm, emission peak 655 nm, green) (Fig. 2Aa,b). GFAP/QD655 signal was amplified by the avidin–biotin complex. Fluorescent signals from QD565 and QD655 were detectable at any depth of the floating section (Fig. 2Aa). The sections were subsequently gold enhanced, which visualized the target lesions (NFTs and astrocytes) on bright-field LM to guide trimming for EM preparation (Fig. 2Ac,d). Gold-enhanced QD565 appeared spherical (Fig. 2Ag,i), while rod-shaped QD655 exhibited accumulation of gold particle only at the tips (Fig. 2Ah,j), which allowed clear distinction even after gold enhancement. The gold enhanced labeling replicated its fluorescence counterpart (Fig. 2Aa vs. c, b vs. d, Ba vs. b). The morphological differences between enhanced QD565 and enhanced QD655 corresponded to respective fluorescent signals, which corroborated the specificity of enhanced QD labeling. Although the ultrastructural preservation of the organelles is compromised by the use of formalin-fixed human autopsy brains, the tau (AT8) -positive fibers were clearly delineated, and immunolabeling was intense (Supplementary Fig. 2A, B). Similar QD655 enhancement was observed in single labeling of Lewy bodies with anti-ubiquitin antibody/QD655 (Fig. 2B), where gold-enhanced QD655 labeled numerous randomly oriented ubiquitin-positive fibers filling the neuron (Fig. 2Bd,e, supplementary Fig. 2C).

We also tried to see if gold-enhanced labeling could be applied to double-label CLEM with Alexa488-labeled 1.4 nm nanogold and QD565 (Supplementary Fig. 3). However, the penetration of Alexa488-labeled 1.4 nm nanogold was limited to less than half of the observed area (Supplementary Fig. 3Ba–c, cross sections, pseudocolored green), even though the diameter of nanogold was smaller than that of QD565. In contrast, QD565 penetrated the entire depth of the sections (Supplementary Fig. 3Ba,b, cross sections, pseudocolored magenta).

In summary, we have successfully used gold-enhanced, double-labeling QDs to compare the characteristics of LM-EM in different cells (neurons with NFTs, Lewy bodies and astrocytes) in the human brain.

## Discussion

Here we proposed a new approach for double-labeling CLEM using different QDs with gold enhancement. QD labeling, initially captured by fluorescence, was enhanced with gold before embedding in epoxy resin. Because the gold-enhanced signals were visible under bright-field microscopy (Fig. 2Ac–e, Supplementary Fig. 3 Ad,e, Bd–f, Supplementary Fig. 4 Ac,e,f, Bb), final trimming of the section was now possible under visual guidance of the targets, which preclude the need of landmarking by microlaser. Enhanced electron density of QDs facilitated their recognition on EM (Fig. 2Ai,j, Be, Supplementary Fig. 2A–C, Supplementary Fig. 3Bj,k, C, Supplementary Fig. 4Be), which preclude the need of EDX analysis. Furthermore, gold enhancement of rod-shaped QD655 on EM grid was accentuated on their tips while spherical QD565 was gold-enhanced as sphere in contrast (Fig. 1). This EM distinction was evident on histological sections where QD565 (green fluorescence) and QD655 (red fluorescence) were used as a reporter pair for double immunolabeling (Fig. 2). The specificity of the labeling of the different QDs was mutually corroborated by LM/EM, since each fluorescence signal corresponded to the EM counterpart (Fig. 2). Furthermore, the use of QDs allowed good penetration of target labeling (Fig. 2Aa, Supplementary Fig. 3Ba,b). The superior tissue permeability of ODs relative to nanogold (Supplementary Fig. 3Ba,b) was compatible with the previous report^11^. This method may allow to immunolocalize different epitopes in relation to ultrastructure of the lesions pre-identified with LM, and pave the way to clarify molecular mechanisms that may drive ultrastructuralization of different proteins with specific reference to its LM counterpart.

In general, CLEM is a very complex procedure that sometimes requires special equipment [reviewed in^29^], modification and development of resin embedding, landmark markers, and genetic tags^30–38^. However, use of QDs allows good penetration of target labeling. Subsequent gold enhancement, which add color to QD labeling, greatly facilitated to trim EM samples containing LM-identified lesions as desired. During the EM preparation, one is quite sure that this trimmed EM block contains the QD-stained targets without doubt, which facilitate laborious work to obtain the final ultrathin section. LM-oriented trimming and comparison between LM-EM images were also facilitated by a virtual slide system that digitalized fluorescence and bright-field images at whatever magnifications of whichever areas desired (Supplementary Fig. 3Aa–c). After introduction of the gold enhancement, correlation between LM-EM images was almost always successful in our laboratory (Fig. 2, Supplementary Figs. 3, 4).

The gold enhancement was not uniform among different QDs. This gold enhancement was selectively accentuated at the tips of the rod-shaped QDs while spherical QDs were homogeneously enhanced. This difference allowed us to distinguish different QDs (Fig. 1) even after gold enhancement. The gold deposition at the tips of the rod-shaped QDs was similar to the previously reported hybrid structure of CdSe and gold^39–41^. However, we found that gold particles can be deposited on QDs by a standard gold enhancement procedure on a tissue section (Fig. 2, Supplementary Fig. 3, 4) or an EM grid (Fig. 1) with autometallographic gold enhancer which catalytically deposits gold ions in solution onto the nanoparticle as metallic gold^42^ (Fig. 1Aa), or even with gold chloride solution only (Fig. 1Aa, b), although the coloring of the label was much weaker with the latter. Although the size of nanogold deposition on QDs initially increased with EBI, it was gradually stabilized (Fig. 1C, Supplementary Fig. 1B, Supplementary video). Because this discovery was carefully confirmed initially with EDX analyses (Fig. 1Bd–i, Supplementary Fig. 1C, Supplementary Fig. 3Bm,o,p), it is now possible to simply enhance different QDs on histological sections so that subsequent trimming and EM recognition of different QD labeling are greatly facilitated without special equipment. Because all reagents used in this study is commercially available, it is possible for every laboratory with conventional transmission electron microscope (TEM) to perform this double labeling QD CLEM with gold enhancement.

The execution of this study's protocol is not without difficulties, as skilled histopathology and EM sample preparation techniques are essential, but the high visibility of the targets after gold enhancement makes it technically very easy to ensure that targets observed with fluorescence microscopy are captured in the EM samples.

In neurodegenerative diseases, intracellular inclusions, in which disease-specific abnormal proteins accumulate in neurons and glial cells in fibrous structures, are often a pathologic feature^22–27,43^. A broad spectrum of clinicopathologic features is known to exist beyond the typical cases. In the pathological examination of autopsy brains, the diagnosis is based on a combination of gross examination and histological findings in representative areas. The feedback of the pathological diagnosis to the clinical setting leads to a more accurate clinical diagnosis. Poor fixation of autopsy brains leads to irreversible tissue damage in subsequent tissue processing and microtomy, and loss of ultrastrutural details and antigenicity in immunohistochemistry. For this reason, formalin fixation of adult human brains is usually performed as a standard over an extended period of time^44^. Whole brain fixation has been shown to be useful in improving the diagnostic yield of neurological diseases, since neurological diseases may be missed in unfixed brains^45^.

Animal models and cellular experiments have provided a detailed picture of how abnormal protein deposits are generated in neurons^46,47^. Because many neurodegenerative diseases are sporadic and present with human-specific pathology, experimental findings need to be validated in human autopsy brains. In human autopsy brains, post-mortem sampling before formalin fixation can be used for CLEM to observe the relationship between organellae and abnormal structures in detail, provided that the target to be observed and its location can be determined in advance^48^. However, most of the CLEM methods developed to date are based on the premise that the object to be observed must be securely contained within a relatively small biopsy area of about 1 mm^3^, and the general process of brain pathology, in which the object is searched for from the whole brain and the ROI is defined, is not well envisioned. It is difficult to extract and observe sparse lesions that can be finally observed by whole-brain search using random biopsy before fixation. Therefore, it is sometimes necessary to have the option of performing EM from the post-fixed brain, even if not under the best conditions. The limitation of this technique is that autopsied brains that have undergone prolonged formalin fixation are not the best conditions for EM because of possible loss of ultrastructure such as intracellular organellae. However, if the CLEM method exists as an option, in which abnormal lesions are identified by extensive fluorescence observation, any lesion is freely selected as the target, and the same lesion is observed by EM, the fiber structure of abnormal proteins in any lesion can be observed with much higher resolution than with fluorescence microscopy. The resolution of EM may make it possible to observe the accumulation of various pathological proteins in neurons that are not visible by fluorescence microscopy, such as α-synuclein positive fibers (5–10 nm in diameter) in Lewy body disease^49^, tau positive fibers (10 nm in diameter) in neurofibrillary changes in Alzheimer's disease^50^, and TDP-43 positive fibers (10–17 nm in diameter)^51^. CLEM of brain pathology would allow more information on rare diseases to be returned to clinical practice from individual autopsy brains. Similar protocol to this study has been demonstrated on mouse brain samples fixed with 4% paraformaldehyde for a relatively short period of time^52^. Preservation of ultrastructure was enhanced when mouse brain samples were used. Although the label used was gold-enhanced nanogold, the processing of the sections was similar, indicating that the technique reported here can better preserve ultrastructure under conditions more suitable for EM.

History of immunoEM has been a never-ending struggle how to compromise conflicting integrities between ultrastructures and epitopes. Use of human brain samples, usually fixed in formalin, is an insurmountable disadvantage for immuno EM, especially when compared with ideally prepared tissues from experimental animals. Because there is no method that satisfies both, how to balance the ultrastructures and immunolabeling the epitopes is a practical issue to be customized according to the samples and the targets. Double-labeling CLEM with different QDs and their gold enhancement, as we established in this study, is a simple and feasible strategy without special equipments. Because this is successfully applicable in human brain samples routinely fixed in formalin, this will pave the way to examine how different molecules are woven into complex ultrastructures to orchestrate their functions in situ.

## Supplementary Information

Supplementary Figures.

Supplementary Video 1.

## Data Availability

Raw image data and all other data that support the findings of this study are available from the corresponding author upon reasonable request.
