# Supplementary material for: Parallel gold enhancement of quantum dots 565/655 for double-labelling correlative light and electron microscopy on human autopsied samples
Source: Sci Rep. 2022 Apr 12;12:6113. doi: 10.1038/s41598-022-09849-8 (PMC9005520; doi:10.1038/s41598-022-09849-8)
Supplement: Supplementary file 1 — Supplementary Figures. [file 41598_2022_9849_MOESM1_ESM.pdf]

Supplementary Fig. 1 | Changes of QD particles by gold enhancement detected by STEM observation and EDX mapping.

A) Changes in QD705 particles after gold enhancement for 1 sec and 5 min using GoldEnhance EM plus®. High contrast nanospheres were developed at the tips of rod-shaped QD705 particles after gold enhancement. On grids with rod-shaped QDs (QD655 and QD705), large gold deposits (approx. 20 nm in diameter) developed occasionally after prolonged incubation with gold enhancer (Supplementary Fig. 1A), while not on grids with QD565. Bars: 20 nm.

B) Progressive expansion of high contrast area on QD705 particles after gold enhancement and subsequent electron beam irradiation (EBI). Fine high-contrast particles of gold-sensitized QD705 (highlighted and pseudocolored yellow) were not gathered from the background but gathered from inside to one end of the nanorod itself by continued EBI, and eventually formed a nano-sized mass at the tip. Bars: 10 nm.

C) EDX mapping of the particles of QD565 (gold enhanced), native QD705, and QD705 (gold enhanced), on nickel grids. Bars: 5 nm.

Supplementary FIG 1

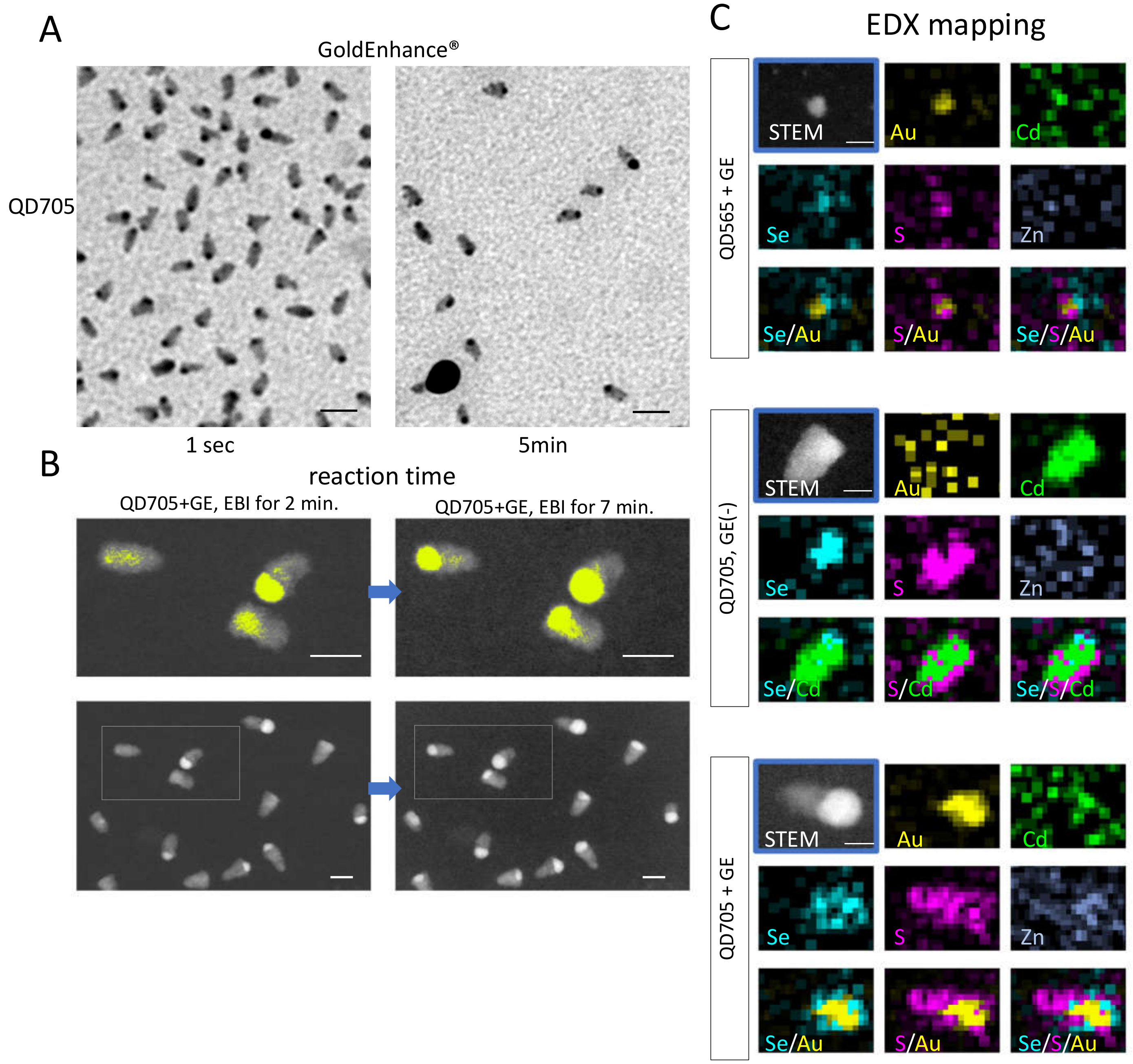

Supplementary Fig. 2 | The electron micrograph of a neurofibrillary tangle immunolabeled for AT8/QD565 and a Lewy body immunolabeled for ubiquitin/ QD655 in formalin-fixed floating section.

Details of the electron micrograph of a neurofibrillary tangle and Lewy body shown in Fig.2A and 2B, respectively. **A-B** A neurofibrillary tangle is immunolabeled with AT8 / gold-enhanced QD565. The fine structure of tau-positive fibers is preserved and immunolabeling was intense. The twisted fibers with a diameter of about 10 nm having a constriction every 80 nm are observed. **C** A Lewy body is immunolabeled with ubiquitin/gold-enhanced QD655. Randomly oriented ubiquitin-positive fibers are seen.

Supplementary FIG 2A

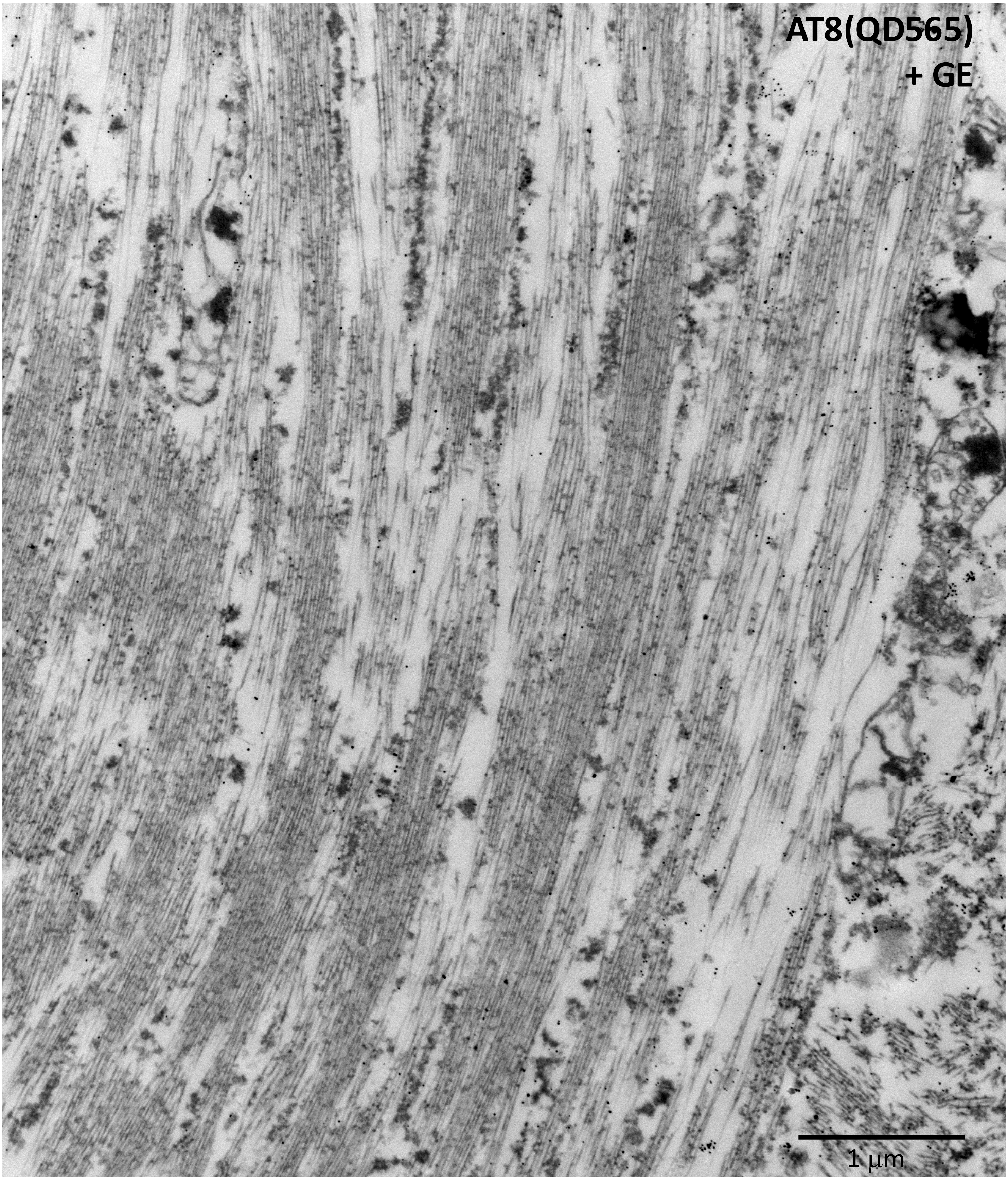

Supplementary FIG 2 B

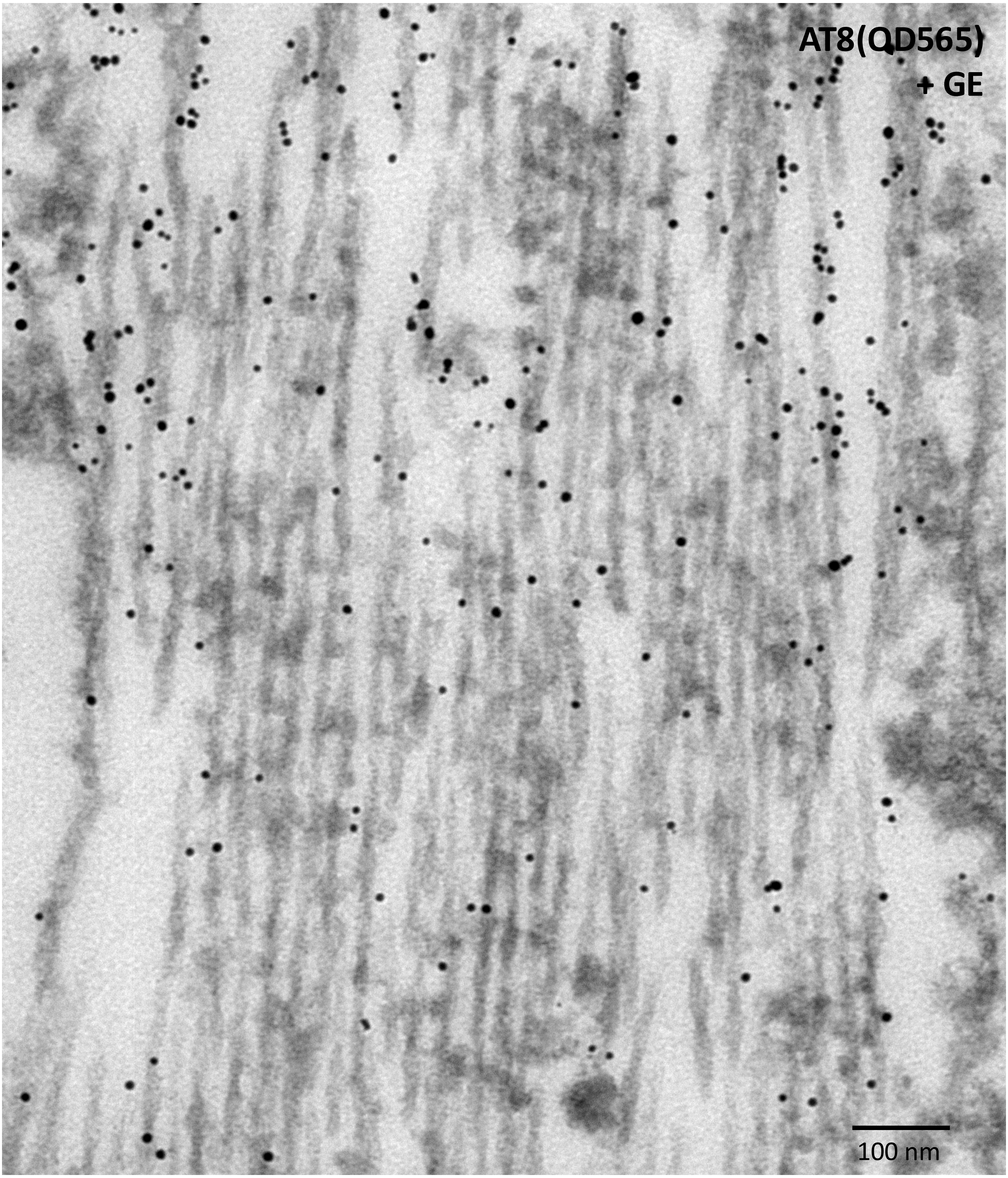

Supplementary FIG 2 C

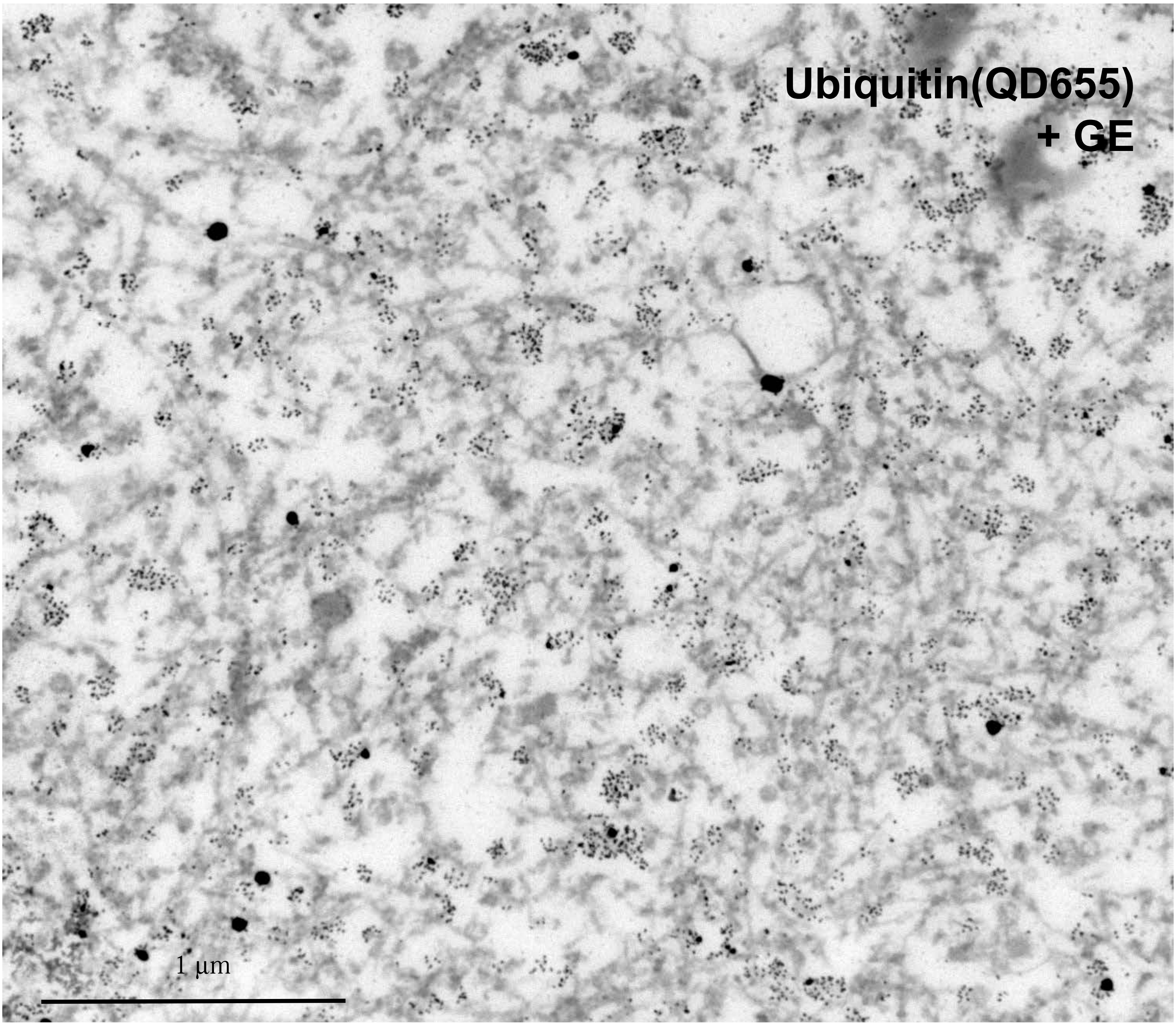

Suplementary Fig. 3 | Dual comparison of light microscopy (LM) and electron microscopy (EM) of the brain tissue double immunolabeled for AT8/QD565 and GFAP/Alexa 488-1.4 nm nanogold: Gold enhancement facilitated trimming EM section and EM recognition of QD/nanogold.

To see if gold enhancement could be applied to double labeling CLEM with QD and nanogold, we double-labeled 26 μm-thick floating sections of formalin-fixed temporal lobe from an autopsied brain (an 80-year-old male, Braak's NFT stage III / neuritic plaque score A, with concomitant Lewy body pathology, supplementary Table 1) [8,13], using anti-phospho-tau antibody (AT8) / QD565 (CdSe-ZnS nanosphere, diameter ~5 nm, emission peak 565 nm, pseudocolored magenta), and anti-glial fibrillary acidic protein (GFAP) antibody / Alexa488-conjugated nanogold (gold nanosphere, diameter 1.4 nm, emission peak 488 nm, pseudocolored green). We targeted a tangle with a small dense part that is suggestive of a fibrillar structure while almost all of the other parts are faintly stained and have no obvious fibrillar structure on fluorescent LM Supplementary Fig. 3 A **c**, arrowhead). We also targeted a mature NFT with flame-like tau filaments (Supplementary Fig. 3A **c**, thick arrow), and a nearby astrocyte (Supplementary Fig. 3A **c**, thin arrow) on fluorescence LM. Detailed fluorescence images of these targets were digitally recorded with the virtual slide system (VS-120, Olympus, Tokyo, Japan, 1376 pixels (horizontal) × 1038 pixels (vertical), at 0.323 μm/pixel with 20 times objective lens and 0.107 μm/pixel with 60 times objective lens) (Supplementary Fig. 3B **a**, **b**, **c**, respectively). Attempt to enhance particles with gold solutions to facilitate their recognition resulted in the light-microscopic visualization of QD565-labeled lesions in parallel with nanogold on bright field (Supplementary Fig. 3A **d**). Since the coloring of gold-enhanced QD labeling was resistant to post fixation with OsO_4 followed by embedding in epoxy resin (Supplementary Fig. 3A, **d**, **e**, enlarged in B **d**, **e**, **f**), it was possible to trim the target area containing the fluorescently-identified lesions under bright-filed stereomicroscopy. Because the gold enhanced labeling replicated its fluorescence counterpart (Supplementary Fig. 3A **c** *vs.* **d**, B **a** *vs.* **d**, **b** *vs.* **e**, **c** *vs.* **f**), this QD labeling remained specific even after the gold enhancement. As expected, the gold-enhanced QD565 particles on tau fibrils showed uniformly round electron dense deposits and their EM contrast was much higher than non-enhanced native QD particles (Supplementary Fig 3B **j,** enlarged in supplementary Fig. 3C, Supplementary Fig. 3B **k**). Subsequent energy dispersive X-ray spectroscopy (EDX) analysis confirmed that the gold-enhanced QD565 particles were composed mainly of gold (Supplementary Fig. 3B **m**, **o**, **p**). Upon comparison between LM and EM, faintly stained part of the tangle shown in Supplementary Fig. 3B **a** contained discontinuous and randomly oriented short fibers, occupying the large part of the cell (Supplementary Fig. 3B **g**, **j**). In contrast, LM-

identified mature NFT with flame-shaped tau deposits (Supplementary Fig. 3B **b**, **e**) contained continuous and aligned long helical filaments (Supplementary Fig. 3B **h**, **k**). Enhanced nanogold particles were distinct from enhanced QDs in that nanogold particles were greatly increased in size to tens of nanometers and non-spherical (Supplementary Fig. 3B **i**, **l**, supplementary Fig. 4). They labeled glial fibers in GFAP-positive astrocyte, which confirmed specificity of this labeling (Supplementary Fig. 3B **c**, **f**, **i**, **l**). However, as demonstrated on cross-sectional views, penetration of Alexa488-conjugated 1.4 nm nanogold was limited to less than half of the observation range (Supplementary Fig 3B **a**, **b**, **c**, green), even though nanogold labeling was enhanced with avidin-biotin complex, while QD565 penetrated the entire depth of the section (Supplementary Fig. 3B **a**, **b**, magenta). This poorer tissue permeability of nanogold relative to QD was compatible with the previous report [1]. From above, we conclude that double labeling with nanogold and QDs is not easily applicable to double labeling of pathological lesions in the human brain such as NFTs.

Supplementary Fig. 3A) (**a-c**) An autopsied brain section was double immunolabeled for phosphorylated tau (AT8)/QD565 (pseudocolored magenta) and GFAP/Alexa488-1.4 nm nanogold (green). The areas enclosed by rectangles in **a** and **b** correspond to **c**. A premature tangle (△), mature NFT (⇨⇨), and astrocyte (→) were targeted. (**d**) The section was gold enhanced, post-fixed, dehydrated, infiltrated with pure epon, and placed between aclar films. The AT8/QD565 labels (△, ⇨) turned dark in the bright-field LM, as well as GFAP/nanogold label (→) after gold enhancement. (**e**) The section was detached from aclar films and attached onto cylindrical epon block. Using the coloring as a landmark, the targeted premature tangle (△), NFT (⇨) and astrocyte (→) were identified, and target-oriented trimming of was facilitated. Bar in **a**: 2mm, **b**: 500 μm, and **c** through **e**: 50 μm.
B) (**a-c**) Extended-focused LM image (EFI) of (**a**) a premature tangle, (**b**) mature NFT, and (**c**) astrocyte, with cross sectional views (20 μm thick) of the widths indicated with dashed lines. While fluorescence of QD565 (magenta) is detected throughout the observational depth, that of Alexa488-1.4 nm nanogold (green) is limited to less than half of the observational depth. (**d-f**) Corresponding targets to (**a-c**) are colored by gold enhancement and identified in epoxy resin. (**g-l**) These EM images are corresponding exactly to the LM images (**a-c**), after gold enhancement of the section (**d-f**). The areas enclosed by rectangles (small j-l) in the low-magnification EM (**g-i**) correspond to the higher magnification EM (**j-l**). Gold enhanced, high contrast QD565 labels (spherical dots, diameter 5-10 nm) are present on the AT8-positive filaments (**g**, **h**, **j**, **k**). The premature tangle (**g**, **j**) consisted of randomly oriented short filaments on EM. Mature NFT (**h**, **k**) consisted of continuous and aligned long helical filaments on EM. Gold enhanced nanogold labels (large irregular intense dots, arrowheads,

diameter -200 nm) are present on GFAP-positive glial fibers (**i**, △ in **l**). (**m, n**) Energy-dispersive X-ray (EDX) mapping of the areas surrounded by the rectangles in **j** and **l** are shown in **m** and **n**, respectively. Both the gold enhanced QD565 (**m**) and nanogold (**n**) showed gold peaks (pseudocolored yellow). (**o-q**) EDX spot analysis of point 10 in **j** (QD565), point 11 in **k** (QD565) and point 12 in **l** (nanogold) show gold peaks (↓). Bars in **a** through **f**: 10 μm, **g** through **i**: 5 μm, **j** through **l**: 200 nm, **m**:10 nm, **n**: 200 nm.

C) Details of the montage image of electron micrograph of the premature tangle shown in Supplementary Fig. 3Ba is shown. The tangle is immunolabeled with AT8 / QD565. Randomly oriented tau-positive short filaments are labeled with 5-10 nm gold-enhanced QD565. The fine structure of tau-positive fibers and surrounding organelles is preserved.

Supplementary FIG 3

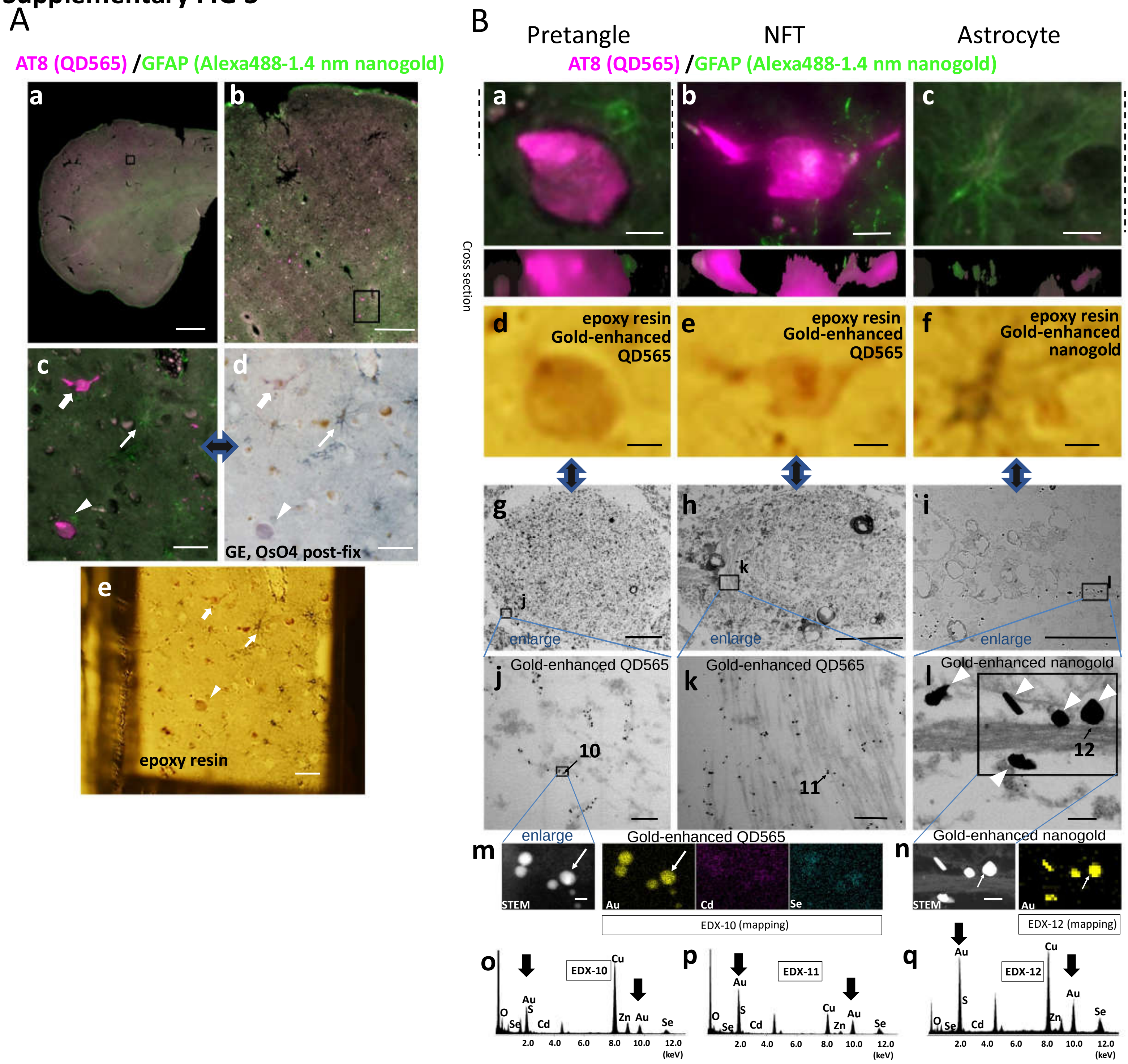

Supplementary FIG 3

C

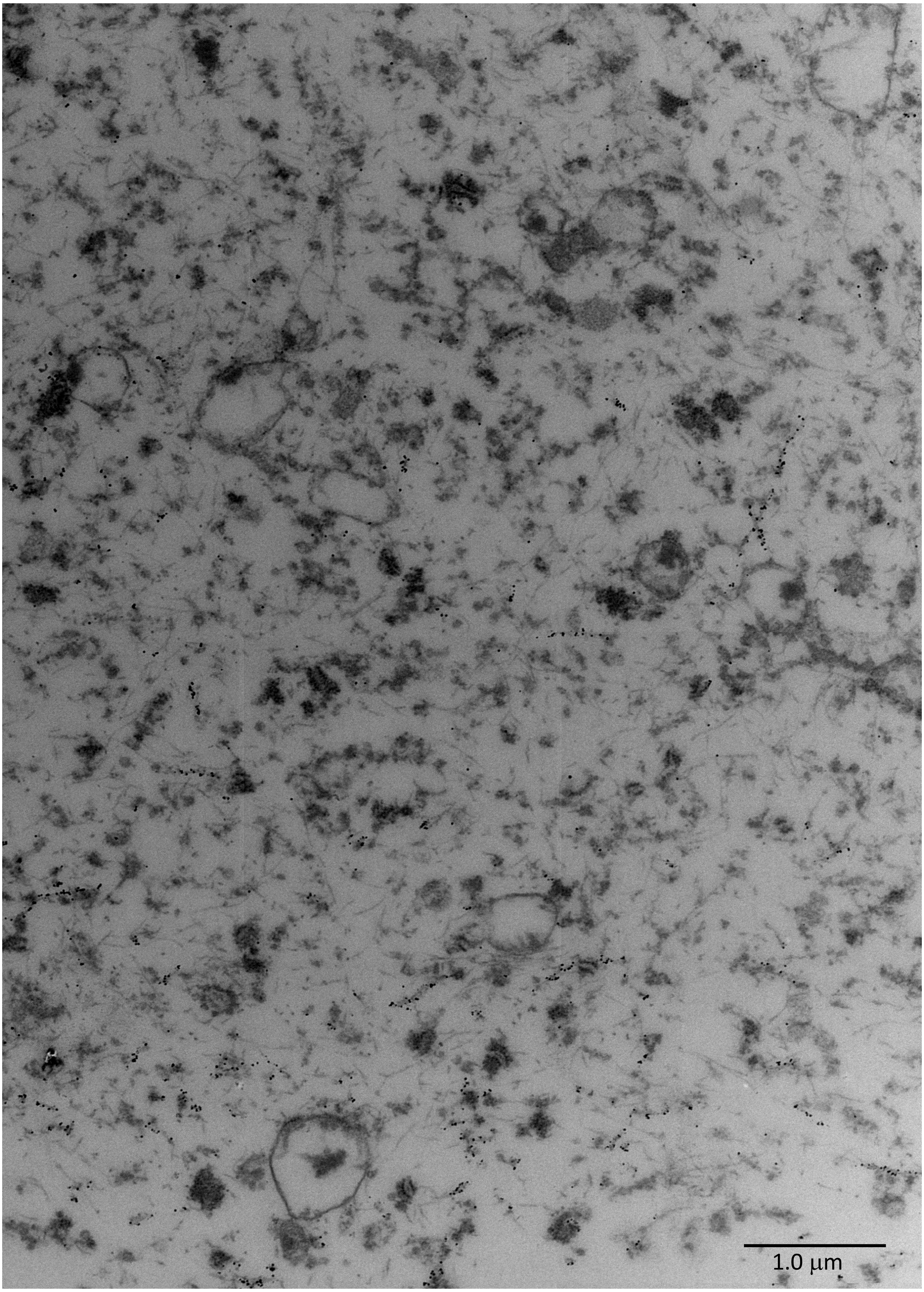

Supplementary Fig. 4 | Dual comparison of bright-field and fluorescence LM images of brain tissue section double immunolabeled for AT8/QD565 and ubiquitin/Alexa488-1.4 nm nanogold before and after gold enhancement.

A) A formalin fixed floating sections of a brain were double immunolabeled for AT8 / QD565 (magenta) and ubiquitin / Alexa488-conjugated 1.4 nm nanogold (green). Colocalization of AT8/QD565 and ubiquitin/Alexa488-conjugated 1.4 nm nanogold is pseudocolored with a mixture of magenta and green. (**a-c**) AT8 and ubiquitin both-positive structure (**a**, ⇨) cannot be identified in the bright-field LM (**b**) because both QD565 and nanogold labels are colorless. However, they become darkened after gold enhancement of the section (**c**). The small arrows (→) indicate the non-labeled neurons around the target (⇨).

d-f) A neurofibrillary tangle that are positive for AT8 and ubiquitin is seen at other site in the same section (**d**, △). After gold enhancement of the section, the QD565 label turned purple in the bright-field LM (**e**). Using this coloring as a landmark, the targeted NFT could be identified in epoxy resin and trimmed to fit in an approximately 0.5 mm square (**f**). Asterisks indicate a nearby capillary. Bars in **a** through **c**: 20 μm, **d** and **e**: 50 μm, **f**: 100 μm.

B) (**a-c**) Extended focused fluorescence LM image (**a**) of the NFT identified in supplementary Fig. 2A **d**, and a bright-field LM image (**b**) and low magnification EM image (**c**) corresponding to image (**a**), after gold enhancement. (**d-f**) The rectangles (small d-f) in the EM images (**c-e**) correspond to the subsequent higher magnification EM images just below. Double labeling for tau/QD565 (small spherical dots, → in **f**) and ubiquitin/nanogold (large intense dots, approx. 40 nm in diameter, △ in **d**, **e**), demonstrated distinct labeling by QD565/tau and nanogold/ubiquitin on paired helical filaments. The gold enhanced QD565 labels showed a gold peak (yellow) in EDX mapping (**f**). Bars in **a** through **c**: 5 μm, **d**: 500 nm, **e**: 100 nm, **f**: 20 nm.

Supplementary FIG 4

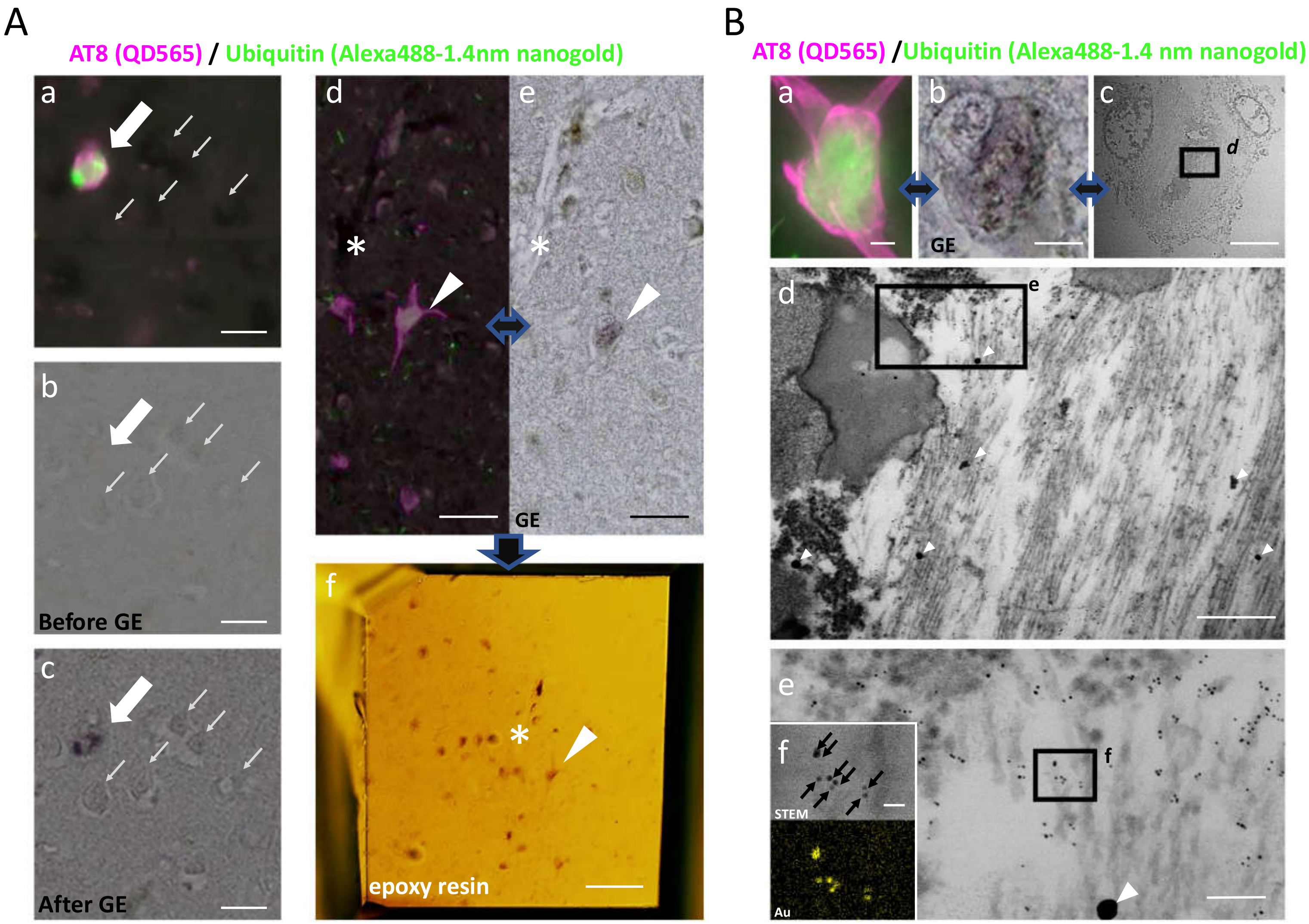

Supplementary Video (separate file)

The EM grid with QD565 and QD655 was observed by STEM (200 kV, × 2,000,000) after gold enhancement. After the start of observation, precipitation of high contrast particle on the QD particles was not evident. However, high contrast nanospheres gradually precipitated on the entire surface of QD565 and at the tip of QD655 nanorods during continuous STEM observation. 2.5 × speed video.

Supplementary Table 1 | Case Information Information on the observed case is presented.

	age at death/gender	agonal state	Fixed brain weight	neuropathological diagnosis		region shown in Fig(s)
				Braak NFT stage	CERAD plaque score	
Case 1 (Fig. 2A,B)	80 y.o. /male	sepsis	1250 g	III	A	temporal lobe
